# Fibroblast-like cells Promote Wound Healing via PD-L1-mediated Inflammation Resolution

**DOI:** 10.7150/ijbs.69890

**Published:** 2022-07-04

**Authors:** Xiao-hui Wang, Wei Guo, Wei Qiu, Luo-quan Ao, Meng-wei Yao, Wei Xing, Yang Yu, Quan Chen, Xiao-feng Wu, Zhan Li, Xue-ting Hu, Xiang Xu

**Affiliations:** 1 Department of Stem Cell & Regenerative Medicine, State Key Laboratory of Trauma, Burn and Combined Injury, Daping Hospital, Army Medical University, Chongqing 400042, P.R. China.; 2 Department of Dermatology, The First Affiliated Hospital of Wenzhou Medical University, Wenzhou 325035, P.R. China.; 3 College of Sericulture, Textile and Biomass Sciences, Southwest University, Chongqing 400715, P.R. China.

**Keywords:** PD-L1, FGF-2, TGF-β1, Fibroblast, Wound healing, Inflammation resolution

## Abstract

Chronic non-healing wounds fail to progress beyond the inflammatory phase, characterized by a disorder of inflammation resolution. PD-1/PD-L1, a major co-inhibitory checkpoint signaling, plays critical roles in tumor immune surveillance and the occurrence of inflammatory or autoimmune diseases, but its roles in wound healing remains unclear. Here, we described a novel function of PD-L1 in fibroblast-like cells as a positive regulator of wound healing. PD-L1 dynamically expressed on the fibroblast-like cells in the granulation tissue during wound healing to form a wound immunosuppressive microenvironment, modulate macrophages polarization from M1-type to M2-type, and initiates resolution of inflammation, finally accelerate wound healing. Loss of PD-L1 delayed wound healing, especially in mice with LPS-induced severe inflammation. Furthermore, the mainly regulatory mechanism is that combination of FGF-2 and TGF-β1 promotes PD-L1 translation in fibroblasts through enhancing the eIF4E availability regulated by both PI3K-AKT-mTOR-4EBP1 and p38-ERK-MNK signaling pathways. Our results reveal the positive role of PD-L1 in wound healing, and provide a new strategy for the treatment of chronic wounds.

## Introduction

Chronic and refractory wounds are still worldwide health problems that concern the physical and mental health of patients and the productivity of society as a whole 1, 2. Wound healing is a series of highly complex biochemical and cellular events that are tightly controlled, divided into 3 concomitant and overlapping phases: inflammation, proliferation, and remodeling 3. The cellular and molecular mechanisms underpinning tissue repair and its failure to heal are still poorly understood, and current therapies are limited. It was estimated that roughly 60% of patients with chronic refractory wound have a recurrence within 1 year after ulcer healing, almost 60% within 3 years, and 65% within 5 years 4, 5. Thus, it is of great scientific significance to further explore the molecular mechanisms of chronic refractory wound.

Chronic wounds after trauma, acute or chronic disease conditions is the consequence of poorly regulated elements of the healthy tissue repair response, including inflammation and associated cellular migration, proliferation, matrix deposition, and tissue remodeling 6. Tissue injury leads to immediate activation of the clotting cascade, and initiate the invasion and recruitment of inflammatory and other tissue repair-related cells. Actually, inflammation plays a key role in the processes of tissue repair and regeneration. The early acute inflammation after injury mobilizes local and systemic defense responses to the site of the wound, and activates tissue repair-related cellular migration, proliferation and matrix deposition 7, 8. However, excessive inflammation inhibits the progression of proliferative phase during wound healing, leading to an impaired restoration of tissue integrity 9. Or a continual chronic inflammation leads to delayed healing 6. It has reported that chronic wound tissue and fluid indicate a continual competition between inflammatory and anti-inflammatory signals leading to a misbalanced environment for proper wound healing to occur, which delays the wound healing 10, 11. Therefore, a more detailed understanding of the mechanisms governing the inflammation and its resolution in the processes of wound healing is needed.

Resolution of acute inflammation is crucial to avoid persistent chronic inflammation and ensure proper return to homeostasis 12. Inadequate or insufficient resolution can lead to chronic inflammation, excessive tissue damage, and dysregulation of tissue healing, leading to fibrosis. However, the mechanisms governing resolution of inflammation remains unclear in the process of wound healing. Various cells, such as neutrophils 13, macrophages 14, 15 has been reported to participated in the regulation of inflammation during wound healing. Recently, Aguirre 16 found that fibroblasts could also regulate inflammation by interacting with T cells in intestinal. However, it remains unclear whether fibroblasts are involved in the regulation of wound inflammation resolution and its fundamental mechanism.

PD-L1 (B7-H1), a crucial immune checkpoint, is not only expressed on cancer cells and hematopoietic cells including T cells, macrophages, dendritic cells (DCs), et al, but also in some non-hematopoietic tissues including pancreas, vascular endothelium, muscle, skin tissues, et al. PD-L1/PD-1 signaling pathway, as a negative regulatory mechanism against the immune response, plays an important role in tumor evasion from immune surveillance and the occurrence of autoimmune diseases 17. However, the role of PD-L1 in inflammation regulation on chronic and refractory wounds is not well defined. In this study, we demonstrated that PD-L1 widely expresses in fibroblast-like cells of wound granulation tissue, and PD-L1 knockdown delays the wound healing. Furthermore, combination FGF-2 with TGF-β1-induced PD-L1 positive fibroblast-like cells are involved in the inflammation resolution of wound healing through regulating the polarization of macrophages from M1 to M2. Altogether, the results of this study uncovered for the first time a key role of fibroblast-like cells in resolution of inflammation and wound healing through expressing PD-L1 induced by FGF-2 and TGF-β1 to promote the polarization of macrophages.

## Materials and Methods

### Isolation of Mouse Embryonic Fibroblasts

Primary mouse embryonic fibroblasts were isolated as previously reported^18^. Briefly, 14 days post fertilization, mouse embryonic fibroblasts were isolated from C57BL/6 embryos and cultured in Dulbecco's modified Eagle's medium supplemented with 10% FBS, 50 units/ml penicillin, and 50 units/ml streptomycin.

### Isolation of mouse macrophages

The mouse peritoneal macrophages were isolated as previously described 19. Briefly, 12 weeks old mice were euthanized with pentobarbital. 5 mL of ice-cold sterile phosphate-buffered saline (PBS) was injected into the peritoneal cavity, followed by aspiration of 4 mL of the cell suspension. After repeating twice, the cells were centrifuged at 200 g for 10 min at 4 °C and then re-suspended in Dulbecco's modified eagle medium (1.0 g/L Glucose, Nacalai Tesque) supplemented with 10% FBS and cultured in a 6-well plate (1 × 10^6^/well). After incubation for 2h, non-adherent cells were removed by gentle washing with serum-free medium and freshly media with 10 % FBS was added for further culturing.

### Animal study

All animal experiments were approved by the Ethics Committee on Animal Experiments of Army Medical University (Chongqing, China). The C57BL/6 background PDL1-/- (PD-L1 KO) mice were purchased from the Jackson Laboratory, (Bar Harbor, ME, USA).

Eight-week-old C57BL/6 mice and PD-L1 KO mice were used to make an excisional wound. The mice were first anesthetized with chloral hydrate, and then removing the hair of the dorsal surface using an electric clipper, followed by a round full-thickness skin excisional wound (0.5 cm in diameter) was made on the back of each mouse. Excessive inflammatory wound was made by subcutaneously injected with LPS derived from K. pneumoniae, 2 and 24 h prior to excisional wounding. Wound was digitally photographed on the day of surgery and every two days thereafter. The wound areas were calculated using ImageJ software (NIH, Bethesda, MD). The results were expressed as the percentage of the original size. The percentage of wound closure was calculated as follows: area of actual wound/area of original wound × 100%. Skin wound biopsy specimens were obtained at days 0, 3, 5, 7 and 9 after injury and snap-frozen and stored at liquid nitrogen for use in the subsequent experiments. For wound cell isolation, wounds were harvested and chopped, then digested with Liberase TL/DNase I (Sigma Aldrich, St. Louis, MO, USA) and plunged through a 100μm nylon filter to yield a single cell suspension.

### Collection of wound exudate

The sponge had been remained on the wound for at least 24 hours and was removed and transferred into a sterile container. The above sponge then been cut into appropriate sizes by sterile scissors and Wound exudates were collected squeezing with a sterile stainless steel garlic press to get the wound exudate. The obtained wound exudate was stored at -20°C for further use.

### Neutralization of FGF-2 and TGF-β1

20 mg/mL anti-FGF-2 neutralizing antibody (Sigma Aldrich, St. Louis, MO, USA) and 50 mg/mL anti-TGFβ1 neutralizing antibody (Thermo Fischer Scientific; former Savant, MA, USA) were incubated along with Wound exudate in FACS, where indicated.

### Co-culture experiments

For our study, we used co-culture models: MEF/MPM. For the co-culture, isolated MPM (1 × 10^6^) were co-incubated with MEF (3 × 10^5^) for 12 h in 3 ml of DMEM supplemented with 10% FBS. Depending on the experiment, MEF were pre-treated with TGF-β1 and FGF-2 for 24h or no stimuli, and then used in the co-cultures with MPM. After co-incubation, both MPM and MEF (harvested by trypsinization) were collected and used for the analysis of the protein expression by flow cytometry. Supernatant was collected and analyzed for cytokine production by ELISA, according to the manufacturer's instructions of the Wuhan Boster Bio-ELISA kit. Values were standardized by measuring total amount of cells and expressed as pg/10^5^ cells. Each group experiment was performed in triplicate.

### Polysome separation and reverse transcription-quantitative polymerase chain reaction (RT-qPCR)

Total RNA was extracted from specimens by TRIzol (Invitrogen, Carlsbad, CA, USA) and then reverse-transcribed using a Revert Aid first-strand cDNA synthesis kit (Takara, Shiga, Japan) according to the manufacturer's instructions. qRT-PCR was performed on the 7500 Real-Time PCR system (Applied Biosystems) using SYBR Premix Ex Taq II (DRR081A, Takara) and normalized to the expression of GAPDH. The relative mRNA expression was estimated as the mean of three replicate assays by 2-ΔΔCt approach. The PCR primer sequences were as follows: PD-L1 sense: 5'-GCTCCAAAGGACTTGTACGTG-3', anti-sense: 5'-TGATCTGAAGGGCAGCATTTC-3'; TNF α sense: 5'-GCTACGACGTGGGCTACAG-3', anti-sense: 5'-CCCTCACACTCAGATCATCTTCT-3'; IL 6 sense: 5'-CTGGAGCCCACCAAGAACGA-3', anti-sense: 5'-GCCTCCGACTTGTGAAGTGGT-3'; GAPDH sense: 5'-GGCATTGCTCTCAATGACAA-3', antisense: 5'-TGTGAGGGAGATGCTCAGTG-3'.

### Additional methods

Immunohistochemistry, Immunofluorescence, Western blotting and Flow cytometry are described in the **Supplementary Materials and Methods**.

### Statistical analysis

Statistical analysis was performed using GraphPad Prism 5. All experiments were repeated no fewer than 3 times. Data is presented as mean ± SEM. The statistical significance of comparisons between two groups was determined with a two-tailed Student's t-test, one-way ANOVA with Kruskal-Wallis multiple comparisons test if more than two treatment groups were compared. P<0.05 indicated statistical significance.

## Results

### Expression of PD-L1 dynamically in the wound granulation tissue

**Figure 1:**
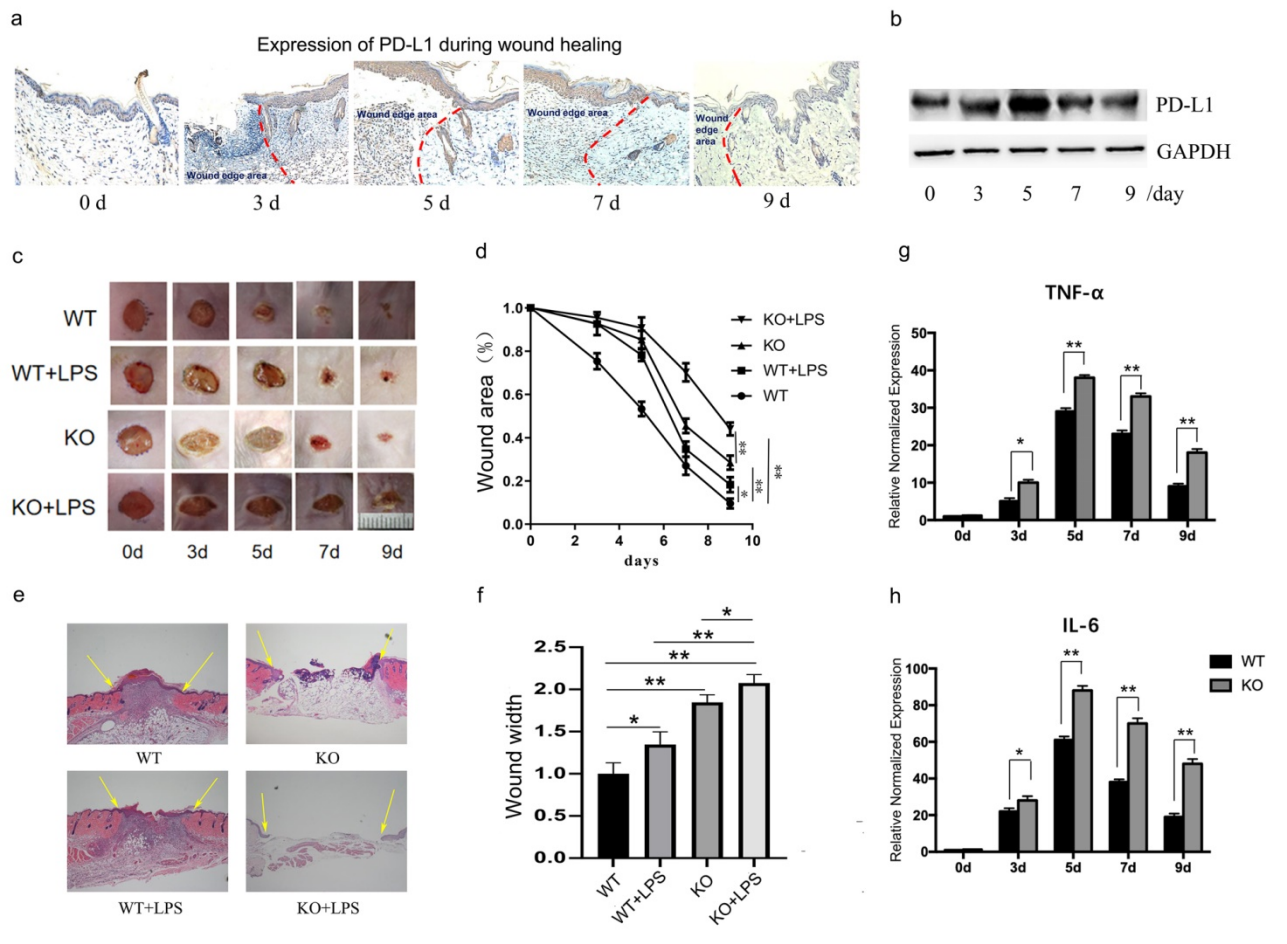
** PD-L1 deficiency prevents wound healing and upregulated inflammatory cytokines.** (a, b)Expression of PD-L1 during wound healing detected by IHC and Western Blot. (c, d)Representative photographs showing macroscopic excisional wound closure of wildtype and PD-L1-/- C57 BL6 mice subcutaneously injected with PBS or 10 μg LPS. (e, f) Representative H&E-stained sections of LPS- or PBS-injected excisional wounds at day 7, arrows indicate wound margins (scale = 500 μm). Analysis of histological wound width reveals LPS or PD-L1-/- significantly delays healing compared with PBS control wounds. n = 5-6 mice/group; (g, h) PD-L1 deficiency up-regulate the expression of TNF and IL-6 during wound healing. *p<0.05, **p < 0.01.

Firstly, we assessed the expression of PD-L1 in the wound granulation tissue. Histological analysis (Fig. 1a) and Western blot results (Fig. 1b and Supplementary Fig. 1a) both showed that the expression of PD-L1 increases gradually at first, and then decreased gradually in the wound granulation tissue. However, interestingly, there was no significant change in the mRNA level of PD-L1 (Supplementary Fig. 1b). These results indicate that PD-L1 may be involved in the resolution of inflammation during early and middle stages of wound healing via a post-transcriptional expression manner.

As we all know, the transition from inflammatory stage to proliferative stage usually occurs 3-5 days after injury. Our results also showed that the expression of inflammatory factors TNF-α and IL-6 peaked on the fifth day after injury in normal mice (Fig. 1g and 1h, WT group). These results suggest that PD-L1 may play an important role in the regulation of inflammation during wound healing.

### PD-L1 promotes wound healing by inhibiting wound inflammatory response

To verify the role of PD-L1 in wound healing, we made an excisional wound in PD-L1 knockout mice. The results showed that wound closure is significantly delayed in PD-L1 knockout mice compared with their WT littermates (Fig. 1c and 1d, WT* vs* KO), especially at day 3, 5 and 7, indicating the positive role of PD-L1 in wound healing.

As indicated by the above results, PD-L1 may be involved in the process of wound healing by participating in inflammation regulation. Actually, the results from excessive inflammatory wound model mice showed that LPS injection delays wound healing in mice, and the loss of PD-L1 aggravated the delayed wound healing caused by LPS stimulation (Fig. 1e and 1f). In addition, compared with wild-type mice, the expression of TNF-α and IL-6 in the wound tissues of PD-L1 knockout mice was significantly increased (Fig. 1g and 1h). These results suggest that excessive inflammatory response actually delays wound healing. Meanwhile, PD-L1 may improve wound healing by inhibiting wound inflammatory response.

### PD-L1 is mainly expressed in fibroblast-like cells of wound tissue, and may regulates macrophage polarization

**Figure 2:**
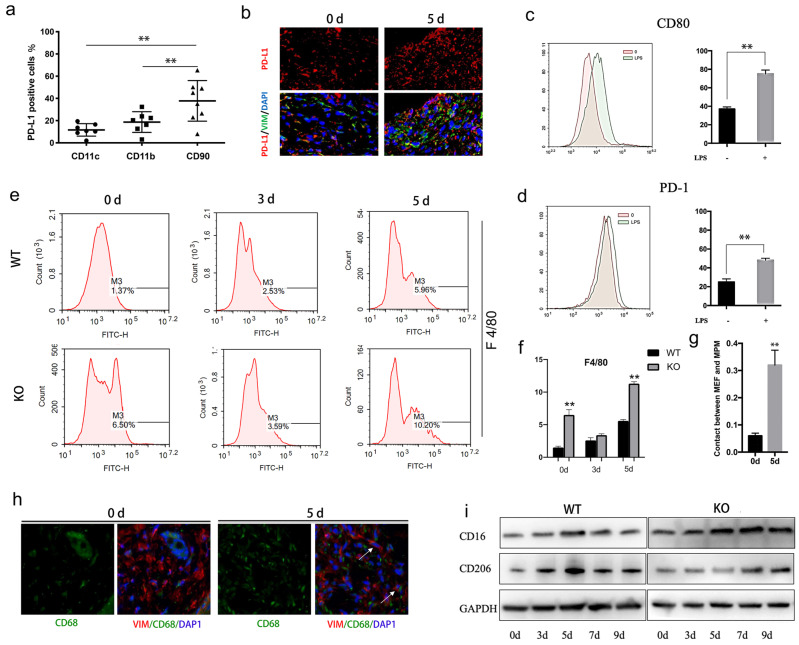
** PD-L1 expressed on fibroblast-like cells regulates macrophages polarization during wound healing.** (a) Expression of PD-L1 in wound tissue cells analyzed by flow cytometry. (n=6). (b) Expresssion of PD-L1(in red) in wound fibroblast-like cells detected by immunofluorescent staining. The fibroblast-like cell population was identified in wound based on positive immunoreactivity for vimentin (VIM). Cell nuclei (in blue) were stained with DAPI. (c, d)Expression of CD80 and PD-1 (green histogram) in LPS treated macrophages analyzed by flow cytometry.(n=4). (e, f) PD-L1 deficiency promotes recruitment of reparative macrophages. (g, h) The fibroblast-like and macrophage cell population was identified in positive immunoreactivity for vimentin(red) and CD68(green), respectively, 0 and 5 days after wound. Cell nuclei (in blue) were stained with DAPI. (i) Expression of CD16(M1) and CD206(M2) during wound healing detected by Western Blot.

The relative distribution of PD-L1 was analyzed by flow cytometry in the cell suspension of wound tissues. Interestingly, the results showed that PD-L1 is mainly expressed in CD90 positive cells (maybe fibroblasts), rather than CD11b and CD11c positive cells (macrophages、DC cells and neutrophils) (Fig. 2a). To confirm that PD-L1 is mainly expressed in fibroblasts during wound healing, we co-stained another fibroblast markers Vimentin and PD-L1 on wound tissue sections. Immunofluorescence analysis confirmed that fewer fibroblast-like cells express PD-L1 at day 0, while a large number of PD-L1 positive fibroblast-like cells are found at day 5 (Fig. 2b).These results indicate that PD-L1 is mainly expressed in fibroblast-like cells during the inflammatory phase of wound healing.

Given the important role of macrophages in wound inflammation, we hypothesized that PD-L1 positive fibroblast-like cells may indirectly regulate inflammation through macrophage. Our results showed that the expression of PD-1 and CD80, two ligands of PD-L1, is increased in LPS-activated macrophages (Fig. 2c and 2d). Furthermore, we also found that, compared to wild-type mice, more macrophages are infiltrated in the wound of PD-L1 KO mice, especially at the 5th day of after injury (Fig. 2e and 2f), and the contact between macrophages and PD-L1 positive fibroblast-like cells is also significantly increased (Fig. 2g and 2h). The results indicate that PD-L1 plays an important role in the recruitment of macrophages to wound area.

Macrophages participate in the regulation of inflammation resolution and wound repair through polarization from M1 to M2. Our results also showed that M1 macrophage marker CD16 gradually decreased from day 5 in wild mice, while the decrease of CD16 was significantly delayed in PD-L1-/- mice (Fig. 2i). In contrast, M2 macrophages marker CD206 showed the opposite change, that is, the expression of CD206 in PD-L1 KO mice was lower than that in wild-type mice (Fig. 2i). These results suggest that PD-L1 may play an important role in macrophage polarization during wound healing.

### TGF-β1 and FGF-2 are key inducers for the expression of PD-L1 in fibroblast-like cells, and the regulation of macrophage polarization

**Figure 3:**
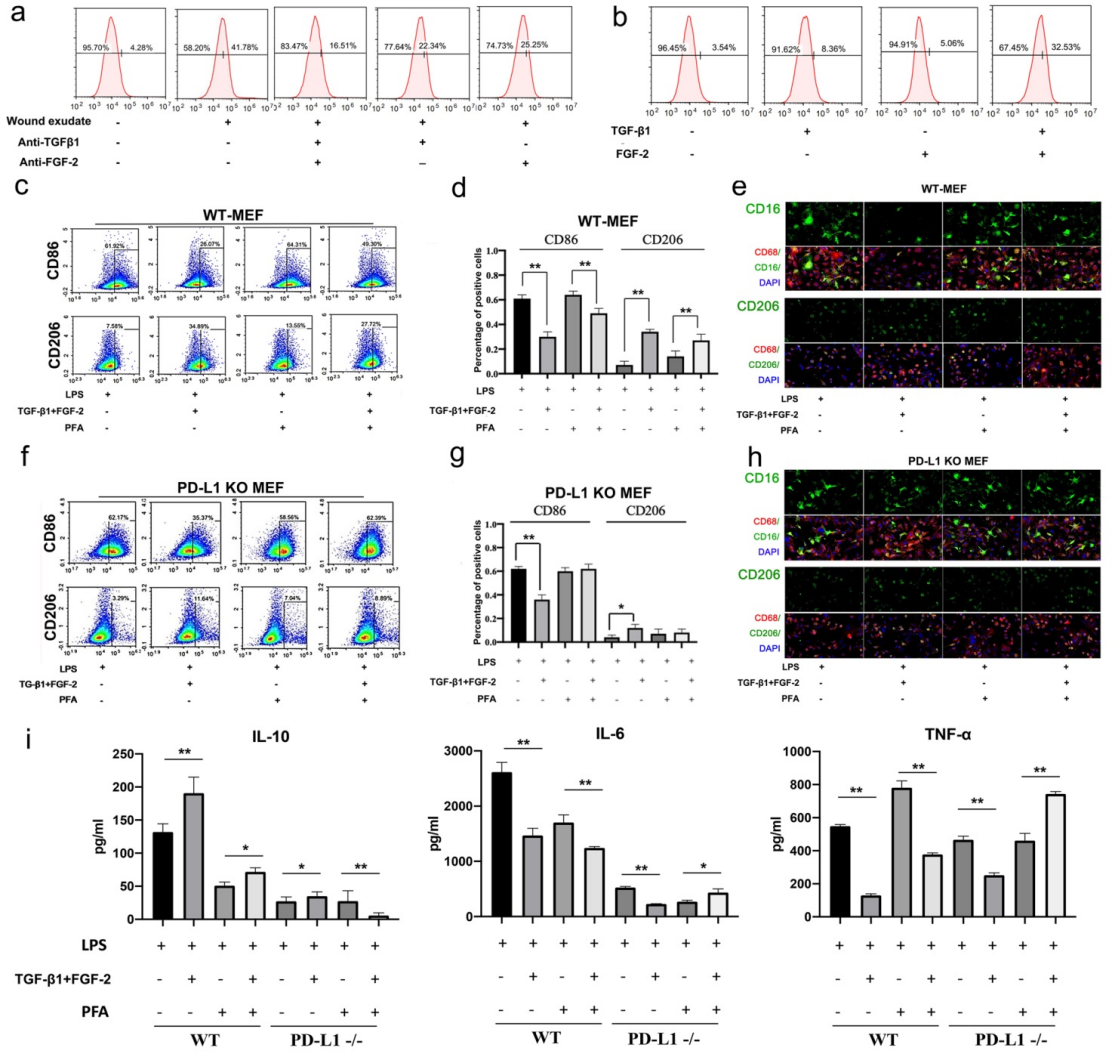
** TGF and FGF up-regulate PD-L1 and skew macrophage polarization**. (a, b) Expression of PD-L1 on the surface of fibroblast-like cells detected by FCM. (c, d, f, g) Expression of CD86 and CD206 in WT-MEF or PD-L1 KO MEF detected by FCM. Macrophages were co-cultured with untreated, or FGF-2 and TGF-β1 pretreated fibroblasts were washed with PBS, blocked in 5% FBS and two color-stained to identify M1 (CD86) and M2 (CD206) macrophage subpopulations.(e, h) Expression of CD16, CD86 and CD206 in macrophages co-cultured with pretreated fibroblast-like cells detected by immunofluorescence staining.(i) Pretreating WT or PD-L1-/- fibroblasts (fixed with Paraformaldehyde or not) affect activated macrophages TNF-α, IL-6 and IL-10 production in a contact-dependent manner. *p < 0.05, **p < 0.01.

In order to clarify the regulation mechanism of PD-L1 expression in fibroblasts during wound healing, we extracted and identified mouse primary fibroblasts (Figure S2) for in vitro experiments. Firstly, we found that wound exudate can induce in vitro the expression of PD-L1 in mouse fibroblast-like cells (Fig. 3a). Furthermore, we found that wound exudate-induced expression of PD-L1 is significantly inhibited by alone or in combination between specific anti-TGF-β1 and anti-FGF-2 antibodies, and combination use of both antibodies displays better inhibitory effect (Fig. 3a). Meanwhile, the expression of PD-L1 in primary fibroblast-like cells is significantly upregulated by combination use of human recombinant TGF-β1 and FGF-2, but only slightly increased by TGF-β1 or FGF-2 factor alone ((Fig. 3b, Supplementary Fig. 3a and 3b). Similar results were found in NIH/3T3 cells (Supplementary Fig. 4a). However, interestingly, consistent with in vivo results, the expression of PD-L1 mRNA in primary fibroblast-like cells is not up-regulated by either TGF-β1 or FGF-2 alone or in combination (Supplementary Fig. 3c).

Furthermore, we in vitro assessed the regulatory function of PD-L1 positive fibroblast-like cells induced by combination of TGF-β1 and FGF-2 on the polarization of LPS-induced macrophages. Our results showed that wild type fibroblast-like cells pretreated with combined TGF-β1 and FGF-2 significantly reduce the expression of inflammatory M1 macrophages marker CD86, but increase the M2 macrophages marker CD206 (Fig. 3c, 3d and 3e). Similarly, PD-L1^-/-^ fibroblast-like cells without paraformaldehyde fixation also displays a same effect as wild type fibroblast-like cells, but a litter weaker. However, PD-L1^-/-^ fibroblast-like cells with paraformaldehyde fixation (excluding the influence of secretory cytokines from fibroblasts) completely lose the regulatory function on the polarization of macrophages from M1-type to M2-type, but wild type fibroblast-like cells with paraformaldehyde fixation still retain some regulatory effect on the polarization of macrophages (Fig. 3f, 3g and 3h). In addition, wild type fibroblast-like cells pretreated with combined TGF-β1 and FGF-2 also significantly reduce the expression of pro-inflammatory factors TNF-α and IL-6, but increase the expression of anti-inflammatory factor IL-10 in co-cultured with LPS-induced macrophages. However, PD-L1^-/-^ fibroblast-like cells with paraformaldehyde fixation completely lost these effects (Fig. 3i). These results indicate that, besides some secretory factors from fibroblast-like cells, fibroblast-like cells pretreated by TGF-β1 and FGF-2 regulate macrophage from M1 to M2 polarization partly through PD-L1 pathway.

### TGF-β1 coordinated with FGF-2 up-regulates the expression of PD-L1 in fibroblast-like cells at the translation level

**Figure 4:**
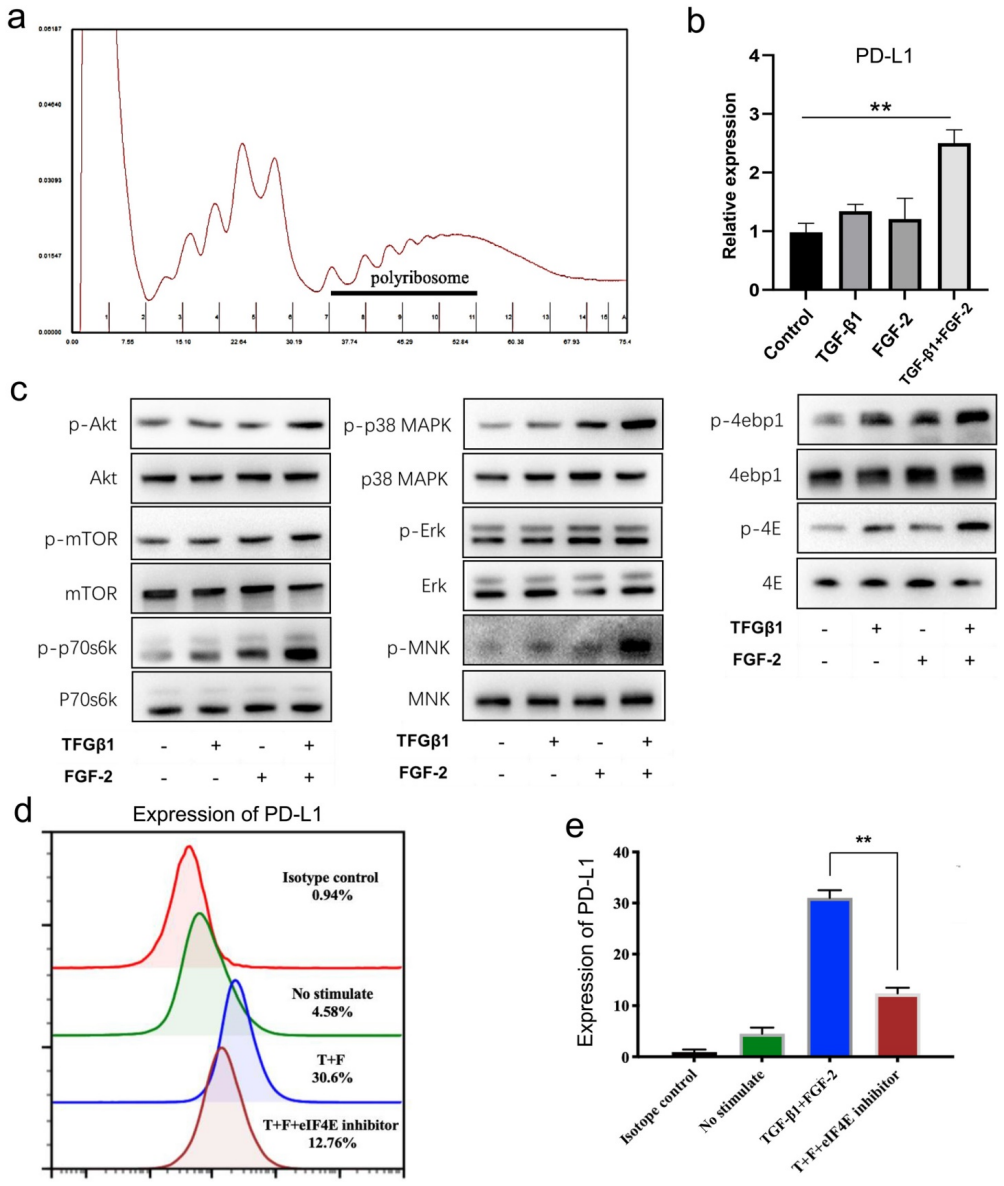
** TGF-β1 together with FGF-2 significantly upregulated fibroblast-like cells PD-L1 at translational levels.** (a, b) Polysome profiles of MEF cells treated 24 h with FGF-2 and TGF-β1. One representative profile from three independent experiments is shown. Percentage of transcripts in each polysomal fraction obtained by sucrose-gradient ultracentrifugation was quantified by qRT-PCR (n = 3). (c) Western blot analysis of the indicated proteins in MEF when stimulated by FGF-2 and TGF-β1. Representative blots from two independent experiments are shown. (d, e) PD-L1 is visualized by flow cytometry in MEF cells stimulated by FGF-2 and TGF-β1 and eIF4E inhibitor. One representative experiment of two is shown. *p < 0.05,**p < 0.01.

From above, we found that the mRNA level of PD-L1 showed no significant changes in wound tissues (Supplementary Fig. 1b) and in fibroblast-like cells in vitro treated with TGF-β1 and FGF-2 either alone or in combination (Supplementary Fig. 3c). Therefore, we furthermore investigated the translational expression of PD-L1 in fibroblast-like cells induced by two cytokines. Polyribosome analysis showed that FGF-2 combined with TGF-β1 significantly increased the mRNA level of PD-L1 in polyribosomes (Fig. 4a, 4b and Supplementary Fig. 4b, 4c), indicating that FGF-2 combined with TGF-β1 actually induces the expression of PD-L1 through translational regulation in fibroblast-like cells.

The formation of eIF4F is a key step in the regulation of mRNA translation. Our results showed that mTOR and p38-ERK-MNK signaling pathway, two key upstream regulatory signaling of the formation of eIF4F, were both significantly activated by combination of FGF-2 and TGF-β1 in fibroblast-like cells, mainly reflected in the significantly up-regulated expressions of p-mTOR, p-p70S6K and p-S6 (Fig. 4c left and Supplementary Fig. 4d), as well as mainly manifested in the up-regulated phosphorylation of ERK, P38 MAPK and MNK (Fig. 4c middle and Supplementary Fig. 4e), respectively. Following above two pathway activation, their two downstream targets, phosphorylated 4EBP1 and phosphorylated eIF4E, were also significantly up-regulated (Fig. 4c right and Supplementary Fig. 4f), indicating a translation activation status in TGF-β1 and FGF-2 treated fibroblast-like cells. Furthermore, 4EGI-1, a competitive eIF4E/eIF4G interaction inhibitor, remarkable impaired the expression of PD-L1 induced by TGF-β1 and FGF-2 in fibroblast-like cells (Fig. 4d, 4e and Supplementary Fig. 4g, 4h). Collectively, these results indicate that combination of FGF-2 and TGF-β1 upregulates the PD-L1 translation of fibroblast-like cells by activating PI3k-AKT-mTOR-4EBP1 and p38-ERK-MNK-eIF4E signaling pathway.

### PD-L1 positive fibroblast-like cells pretreated by TGF-β1 and FGF-2 promote excessive inflammation induced chronic refractory wound healing in PD-L1-/- mice

**Figure 5:**
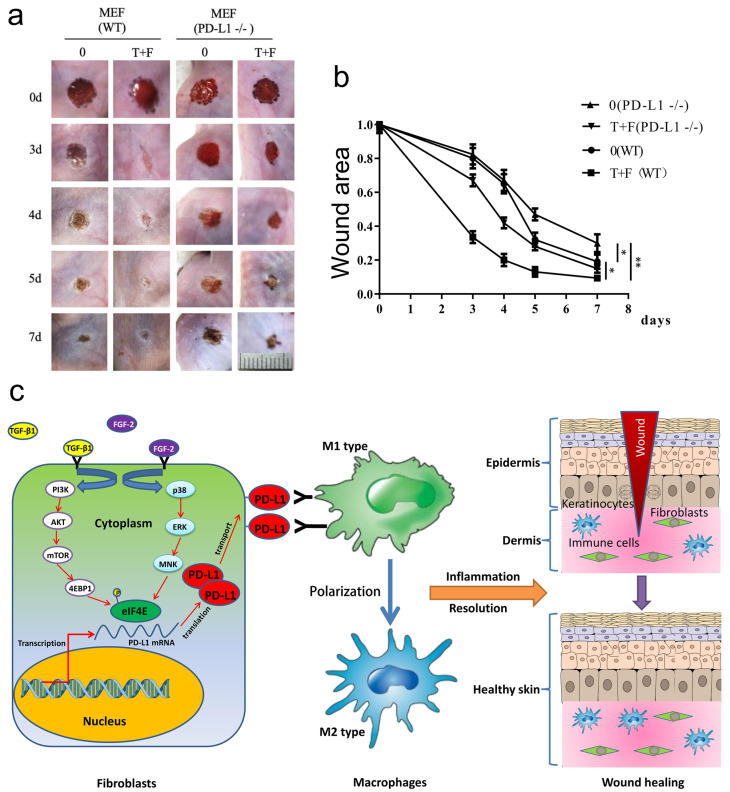
** PD-L1 positive fibroblast-like cells pretreated by TGF-β1 and FGF-2 promote chronic refractory wound healing in vivo.** (a) Representative photographs showing macroscopic excisional wound closure treated with PD-L1 positive and negative MEF in PD-L1-/- C57 BL6 mice. The wild-type and PD-L1-/- mice fibroblast-like cells were isolated and cultured in Dulbecco's modified Eagle's medium (Gibco) supplemented with 10% fetal bovine serum (FBS), 50 units/mL penicillin and 50 units/mL streptomycin (Sigma). Followed by pretreated with TGF-β1 and FGF-2, approximately 1x 10^5^ fibroblast-like cells were further suspended in saline and sprayed over the wound area of the mice. (b) Planimetric analysis of wound photographs reveals significantly delayed at 3-5 days after wounding when PD-L1-/- MEF were used. (c) The schematic diagram of the mechanism of PD-L1 regulating wound healing.

To assess the effectiveness of fibroblast-like cells pretreated by TGF-β1 and FGF-2 on LPS-induced excessive inflammation wound healing in PD-L1-/- mice, the wild-type and PD-L1-/- mice fibroblast-like cells pretreated with combination of TGF-β1 and FGF-2 were transplanted onto the wound area of the mice. Surprisingly, compared with the PD-L1-/- fibroblast-like cells, the transplantation of wild-type fibroblast-like cells pretreated with or without two-factor both significantly accelerated wound healing in PD-L1-/- mice, especially 3 to 5 days after injury. Meanwhile, compared with unpretreated fibroblast-like cells, two-factor-pretreated wild-type and PD-L1-/- fibroblasts both significantly improved wound healing in PD-L1-/- mice (Fig. 5a and 5b). Collectively, these results confirm that the PD-L1 positive fibroblast-like cells plays an important role in wound healing of LPS-induced excessive inflammation mice, and besides PD-L1, other some soluble growth factors or receptors from fibroblast-like cells induced by combination of TGF-β1 and FGF-2 may be also involved in the regulation of wound healing.

## Discussion

Wound healing is a series of highly complex biochemical and cellular events that are tightly controlled 6. As we know, skin fibroblasts are critical in supporting normal wound healing, involved in key processes such as breaking down the fibrin clot, creating new extra cellular matrix (ECM) and collagen structures to support the other cells associated with effective wound healing, as well as contracting the wound 20. Here, we reported a novel function of fibroblast-like cells through up-regulating the expression of PD-L1 to form a wound immunosuppressive microenvironment, modulate macrophages polarization from M1 to M2, and initiate resolution of inflammation, finally accelerate wound healing. Consistent with our results, fibroblasts have been considered as sentinel cells of inflammatory response over the course of tissue repair 21, and display some evolving functions including an early pro-inflammatory, leukocyte-recruiting, and anti-migratory phenotype, then evolving into a proliferative, pro-fibrotic, and pro-angiogenic phenotype, finally an anti-angiogenic homeostatic-like myofibroblast phenotype 22.

PD-1/PD-L1 pathway plays an important role in tumor immune surveillance by regulating T cells activation, exhaustion and infiltration, and its inhibitory strategies has emerged as a significant treatment modality for several cancers. Additionally, PD-1/PD-L1 signaling also plays critical role in the occurrence and development of inflammatory and immune diseases 23. In this study, we first found that PD-L1 dynamically expresses in the wound granulation tissue, and PD-L1 deficiency leads to increased local inflammation and delayed wound healing, especially in LPS-induced excessive inflammation model, and the wound healing time was highly prolonged. These findings support that PD-L1 signal might play critical role in wound healing by regulating resolution of inflammation.

Inflammatory regulation disorder is a major cause for chronic refractory wounds 1. Tissue injury leads to an acute inflammatory response, which restores tissue homeostasis through removing microorganisms and necrotic tissues, and activates tissue repair-related cells 7, 8. The inflammatory response will resolute in an orderly and harmlessly manner following wound tissue repair progresses. However, resolution of inflammation disorder will lead to a continual chronic inflammation, an impaired restoration of tissue integrity and delayed healing 6, 9.Diabetes and age-related skin ulcers are characterized by chronic inflammation, and inhibiting chronic inflammation can accelerate ulcer healing 24, 25. Consistent with these results, we also confirmed that LPS-induced excessive inflammation wounds display delayed healing.

Macrophages play the role of "commander in chief" and rely on functional gene reprogramming to induce an orderly polarization including M1-type macrophages-initiating acute inflammation response and M1-type macrophages-inducing resolution of inflammation, finally promoting wound tissue regeneration 26-28.In this study, we surprise found that fibroblast-like cells promote wound healing in a novel mechanism, dynamically expressing PD-L1 to form a wound immunosuppressive microenvironment, modulate macrophages polarization, and initiate wound resolution of inflammation. However, the detailed mechanism of PD-1/PD-L1 in macrophages polarization is still unclear, more works including specific fibroblasts PD-L1 knock-out mice model need to be performed to understand the role of PD-L1 signal from fibroblasts in macrophages polarization and wound resolution of inflammation in the future.

Many evidences have confirmed that the expression of PD-L1 is regulated by STAT, HIF-1, Myc, and other transcriptional factors 29. Besides, recent studies have explored the post-transcriptional regulatory mechanisms on PD-L1 expression, including Akt/mTOR/S6K1 pathway-mediated PD-L1 protein translation, glycosylation-mediated PD-L1 protein stability, and ubiquitination-mediated PD-L1 protein degradation 29-31. Here, we further investigate the regulatory mechanism of PD-L1 expression in fibroblast-like cells during wound healing, and confirmed that the combination of TGF-β1 and FGF-2, but not alone, up-regulates the expression of PD-L1 in skin fibroblast-like cells during wound healing, suggesting a synergistic effect of two cytokines. Moreover, interestingly, FGF-2 combined with TGF-β1 did not affect the mRNA level of PD-L1, only increased the expression of PD-L1 in fibroblast-like cells at the protein level, suggesting its express regulation might be in post-transcription.

The formation of eukaryotic translation initiation factor complex (eIF4F, formed with eIF4A, eIF4G, and eIF4E) is essential for cap-dependent translation initiation. 4E-BP1, a negative regulator of protein synthesis, is a directly downstream molecular of mTORC1 pathway. The phosphorylated 4E-BP1 prevents its binding to the cap-binding protein eIF4E at 5′-cap of mRNAs and thereby promotes the participation of eIF4E in composition of eIF4F 32. Furthermore, the activity of eIF4E is regulated not only by the Akt/mTOR pathway but also by the MAPK/MAP kinase-interacting kinase-1 (MNK1)-mediated phosphorylation of eIF4E at the serine 209 site 33. Our previous study confirmed that Akt/mTOR pathway and eIF4E both are activated, and enhancing their activation accelerates wound healing 34, 35. In this study, we confirmed that combination of FGF-2 and TGF-β1 upregulates the PD-L1 translation in fibroblast-like cells by activating both PI3k-AKT-mTOR-4EBP1 and p38-ERK-MNK-eIF4E signaling pathway. However, synergistic regulatory mechanism of two cytokines, as well as whether there still are other factors involved in PD-L1 expression regulation is completely unclear, more works need to be done in the future.

In conclusion, we first report a novel function of PD-L1 in fibroblast-like cells as a positive regulator of wound healing. PD-L1 dynamically expresses on the fibroblast-like cells in the wound granulation tissue to form a wound immunosuppressive microenvironment, modulate macrophages polarization from M1-type to M2-type, and initiate resolution of inflammation, finally accelerate wound healing. Moreover, it is a key regulatory mechanism of PD-L1 expression that combination of FGF-2 and TGF-β1 enhances the eIF4E availability by both PI3k-AKT-mTOR-4EBP1 and p38-ERK-MNK signaling pathway (Fig. 5c). Our research deepens people's understanding of the role of immune checkpoint in wound healing and provides new strategy for chronic refractory wound treatment.

## Supplementary Material

Supplementary materials and methods, figures.

## Abbreviations

PD-L1: Programmed death ligand 1
PD-1: Programmed cell death protein1
FGF-2: Fibroblast growth factor 2
TGF-β1: Transforming growth factor beta 1
IL-6/10: Interleukin 6/10
LPS: Lipopolysaccharide
PI3k: Phosphatidylinositol-4,5-bisphosphate 3-kinase
AKT: Protein kinase b
mTOR: Mechanistic target of rapamycin kinase
eIF-4EBP1: Eukaryotic translation initiation factor 4E binding protein 1
ERK: Mitogen-activated protein kinase 1
MNK: Mitogen-activated protein kinase-interacting kinases
eIF4E: Eukaryotic translation initiation factor 4E
