# Supplementary material for: Fibroblast-like cells Promote Wound Healing via PD-L1-mediated Inflammation Resolution
Source: Int J Biol Sci. 2022 Jul 4;18(11):4388–99. doi: 10.7150/ijbs.69890 (PMC9295062; doi:10.7150/ijbs.69890)
Supplement: Supplementary file 1 — Supplementary materials and methods, figures. [file ijbsv18p4388s1.pdf]

Supplementary materials and methods

Immunohistochemistry

The mice were sacrificed at the indicated day after surgery, and complete wound tissues were separated from the back. The paraffin-embedded tissues were subjected to H&E or immunohistochemistry staining according to a standard procedure. Briefly, the slides were incubated with anti-PD-L1 antibody (1:100; Abcam, Cambridge, MA, UK) at 4 °C overnight, followed by incubation with horseradish peroxidase (HRP)-conjugated secondary antibodies for 30-min at room temperature.

Immunofluorescence

After fixation with paraformaldehyde and permeabilized with 0.1 % Triton X-100, mouse peritoneal macrophages or paraffin-embedded wound tissue sections were incubated with the desired primary antibodies obtain from Abcam, Cambridge, MA, UK against the vimentin (1:800), CD68 (1:800;), PD-L1 (1:100), CD16 (1:100), or CD206 (1:800) overnight at 4 ℃ and with secondary antibodies for 60 min at room temperature, Nuclei were counter-staining with DAPI for 5 min. Finally, the cells were examined and photographed with a confocal imaging system (Olympus FV1200).

Western blotting

Tissue or cell samples were washed with ice-cold PBS and lysed in lysis buffer (20 mM Tris (pH 7.5), 150 mM NaCl, 1% Triton X-100, 1 mM 1 mM β-glycerophosphate, 10 mM Na3VO4, and protease inhibitor cocktail tablets (Roche) as previously described (Xu, et al, 2010 & Guo, et al, 2020). The lysate was centrifuged at 12,000 rpm for 15 min at 4 ℃. The concentration of total proteins was determined using a BCA Protein Assay Kit (Thermo

Fisher Scientific). Equal amounts of the extract were subjected by SDS-PAGE and transferred into PVDF membranes, and then blocked with 5% fat-free milk in Tris-buffered saline with 0.1% Tween 20 (TBST) for 60 min. The membranes were incubated overnight at 4 ℃ with specific primary antibodies (1:1000). The primary antibodies were obtained from Cell Signaling Technology, Beverly, MA, USA (P-AKT, AKT, P-mTOR, mTOR, p-p70S6K, p70S6K, p-p38 MAPK, p38 MAPK, p-Erk, Erk, p-MNK, MNK, p-4EBP1, 4EBP1, p-4E, 4E and β-actin), or Abcam, Cambridge, UK (PD-L1,CD16 and CD206). After washed with TBST for three times, the blots were incubated with goat anti-rabbit IgG or goat anti-mouse IgG (1:10,000; Zhongshan Goldenbridge, Beijing, China) for 2h at room temperature. The protein bands were revealed by an ECL luminescent detection system (GE Healthcare, USA). β-Actin was used as a loading control for western blots.

Flow cytometry

Following treatment of co-culture model, cells were detached from the culture flasks by treatment with non-enzymatic cell dissociation buffer (Sigma) at 37 ℃ for 15–30 min, followed by two washes with cold PBS. Immunostaining for mouse CD11c, CD11b, CD90, PD-L1, F4/80, CD86, and CD206 or isotype controls was performed according to a standard eBioscience surface immunostaining flow cytometry protocol. Cells were analyzed by flow cytometry using ACEABIO NovoCyte (ACEA) per the manufacturer’s procedures. Area, height and width parameters for forward and side scatters (FSC and SSC, respectively) were used to discriminate single live cells. Flow cytometry data were analyzed using NovoExpress (ACEA) software.

To identify macrophage M1/M2 populations, two-color staining flow cytometry was performed. The first staining differentiated the macrophage population from the mixed cells, and the second staining identified the M1/M2 macrophages from within the previously-gated population. The primary antibodies used for cell-surface staining and detection by flow cytometry were rat anti-mouse F4/80, rat anti-mouse CD86 and rat anti-mouse CD206, each at 1:100 dilution, and mouse (Ig)G1 isotype (1:100 dilution). CD86 and CD206 are surface markers representative of mouse M1 and M2 phenotypes, respectively. F4/80+/CD86+ cells were considered to be M1 macrophages, whereas F4/80/CD206+ cells were identified as M2 macrophages.

Supplementary results

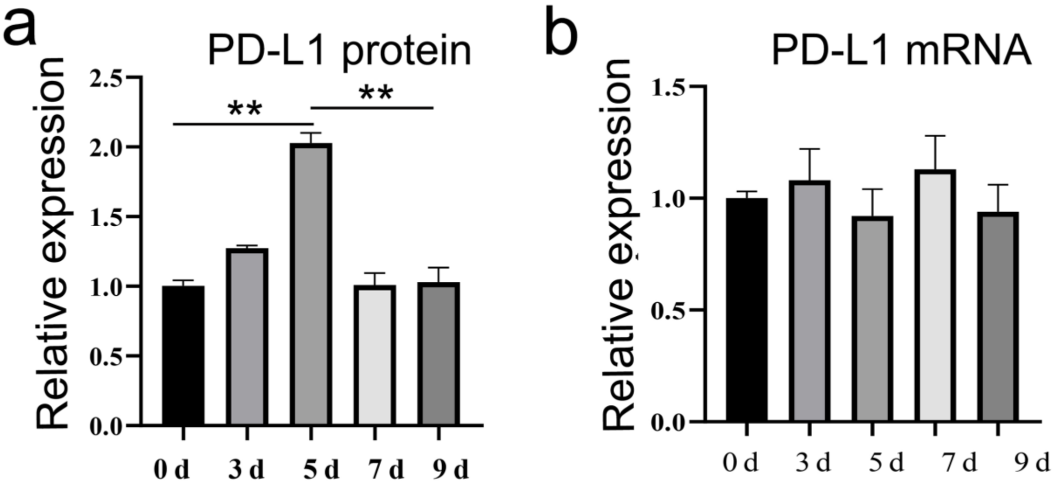

Supplementary figure 1.Expression of PD-L1 during wound healing. (a) Expression of PD-L1 during wound healing analyzed by Western Blot. (b) Expression of PD-L1 during wound healing analyzed by qRT-PCR. *p < 0.05,**p < 0.01.

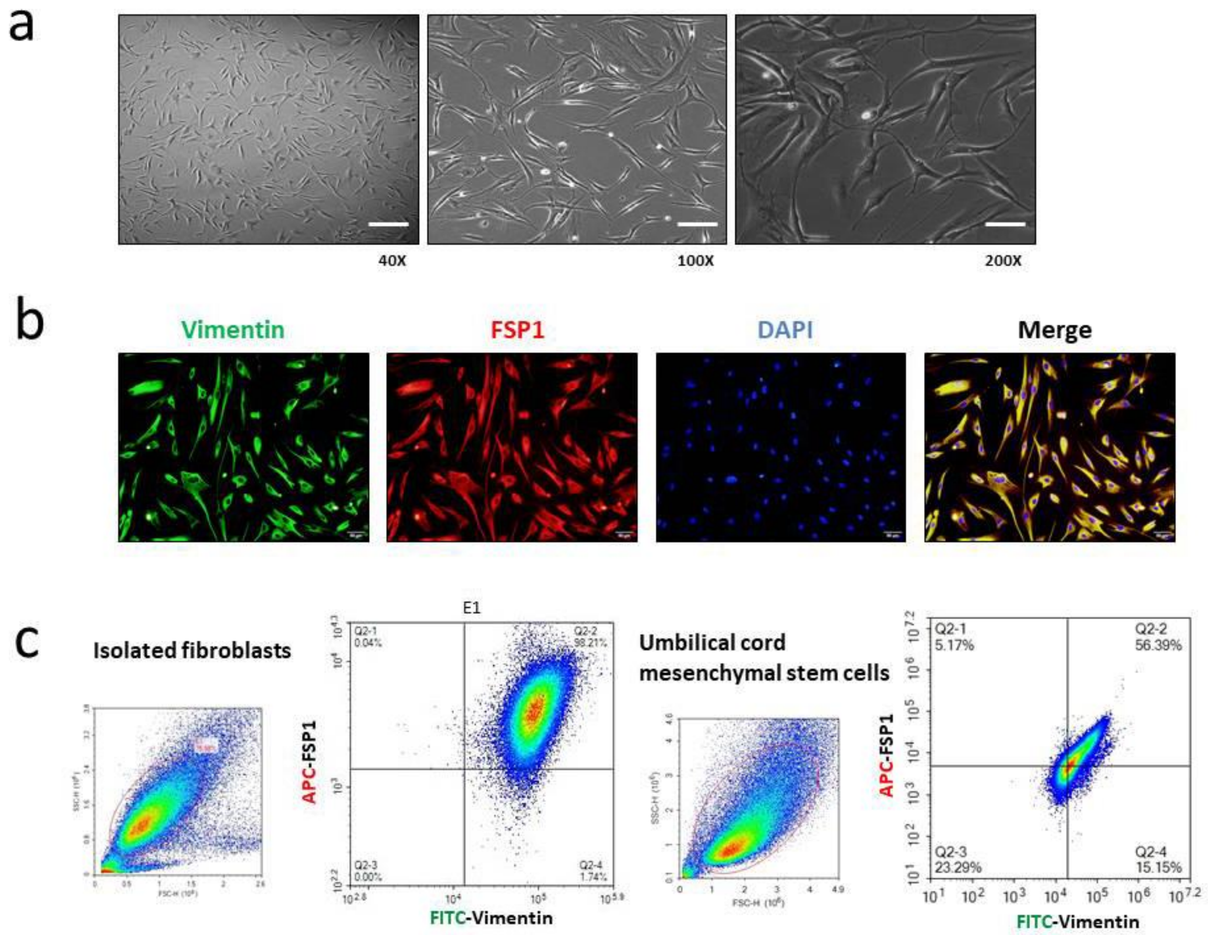

Supplementary figure 2. Identification of isolated fibroblasts. (a) Morphology of isolated fibroblasts. (b) Expression of Vimentin and FSP1 in isolated fibroblasts detected by immunofluorescence. (c) Expression of Vimentin and FSP1 in isolated fibroblasts (Left) and UC-MSCs（Right）detected by FCM.

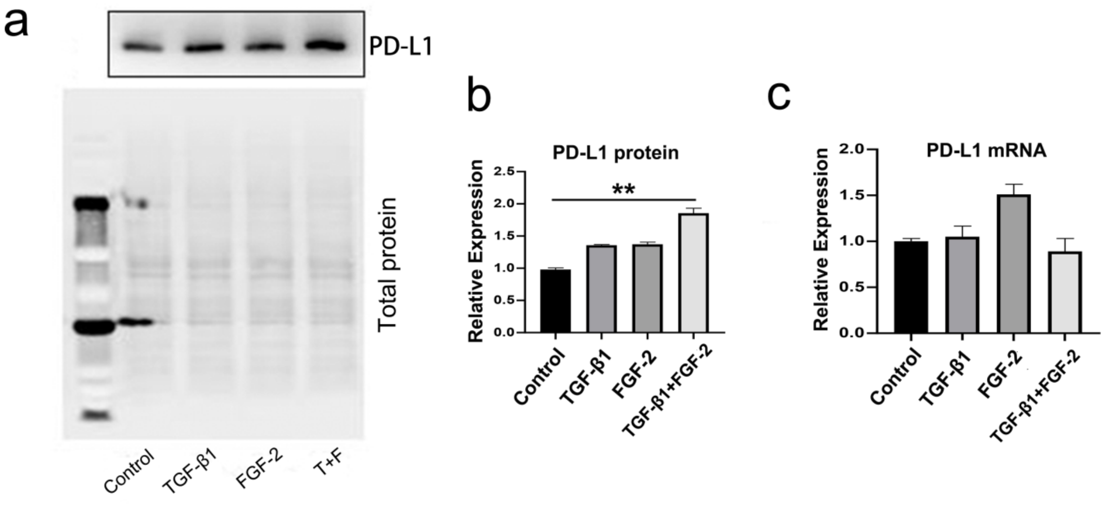

Supplementary figure 3. Expression of PD-L1 in fibroblast-like cells treated by TGF-β1 and FGF-2. (a,b) Expression of PD-L1 in fibroblast-like cells treated by TGF-β1 and FGF-2 analyzed by Western Blot. (c) Expression of PD-L1 in fibroblast-like cells treated by TGF-β1 and FGF-2 analyzed by qRT-PCR. $*p < 0.05$, $**p < 0.01$.

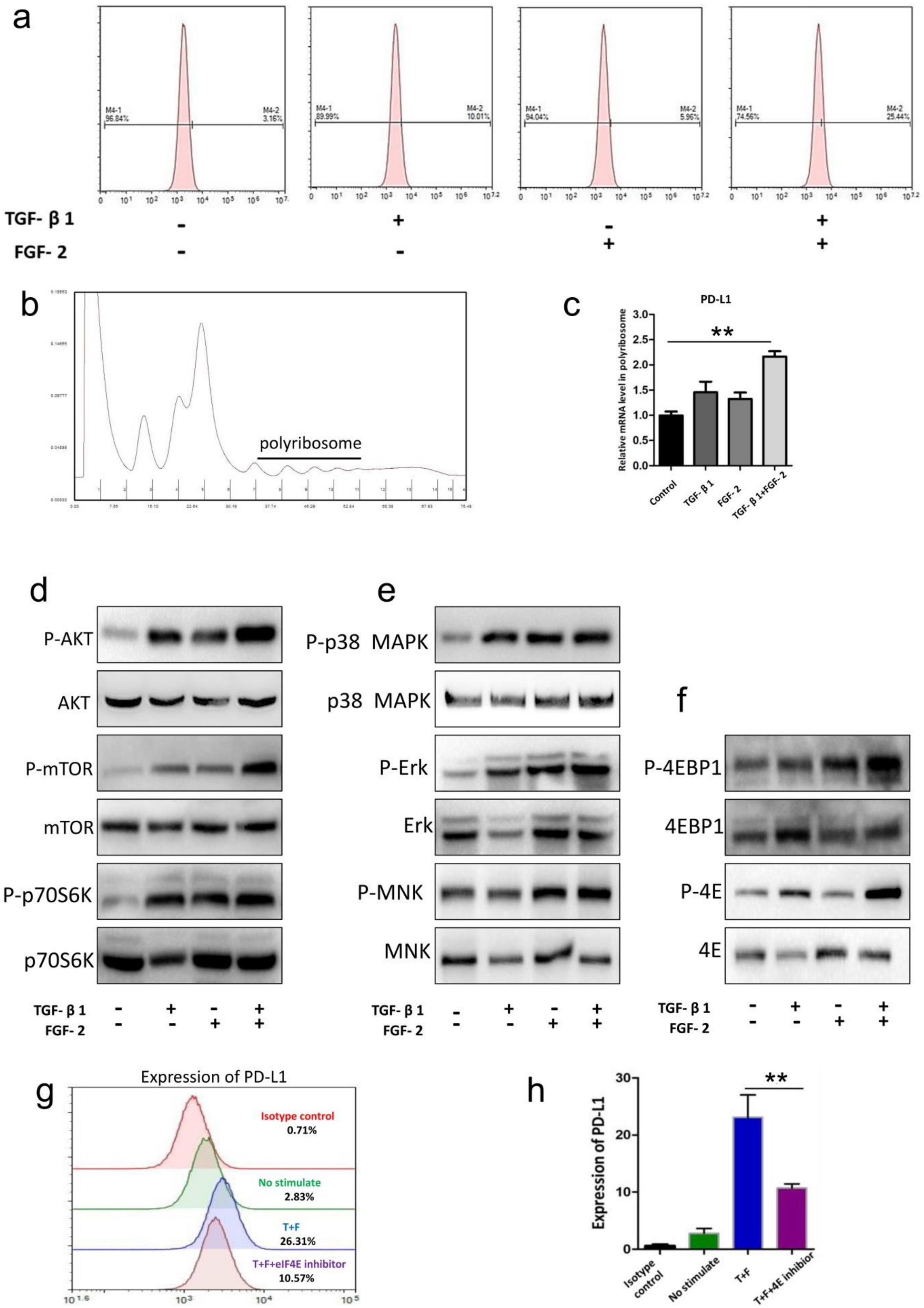

a
M4-1
96.84%
M4-2
3.16%
M4-1
89.99%
M4-2
10.01%
M4-1
94.04%
M4-2
5.96%
M4-1
74.56%
M4-2
25.44%
TGF- β 1
FGF- 2
b
polyribosome
c
PD-L1
Relative mRNA level in polyribosome
**
Control
TGF- β 1
FGF- 2
TGF- β 1+FGF- 2
d
P-AKT
AKT
P-mTOR
mTOR
P-p70S6K
p70S6K
e
P-p38 MAPK
p38 MAPK
P-Erk
Erk
P-MNK
MNK
f
P-4EBP1
4EBP1
P-4E
4E
TGF- β 1
FGF- 2
g
Expression of PD-L1
Isotype control
0.71%
No stimulate
2.83%
T+F
26.31%
T+F+eIF4E inhibitor
10.57%
h
Expression of PD-L1
**
Isotype control
No stimulate
T+F
T+F+4E inhibior

Supplementary figure 4. Effects of TGF-β1 and FGF-2 on NIH/3T3 cells.(a) Expression of PD-L1 on the surface of NIH/3T3 cells detected by FCM. (b,c) Polysome profiles of NIH/3T3 cells treated 24 h with FGF-2 and TGF-β1. (d,e,f) Western blot analysis of the indicated proteins in NIH/3T3 cells when stimulated by FGF-2 and TGF-β1. (g,h) PD-L1 is visualized by flow cytometry in NIH/3T3 cells stimulated by FGF-2 and TGF-β1 and eIF4E inhibitor. *p < 0.05,**p < 0.01.
